# Massachusetts Dental Society

**Published:** 1902-04

**Authors:** 


### MASSACHUSETTS DENTAL SOCIETY.

The thirty-seventh annual meeting of the Massachusetts Dental Society convened at the Massachusetts Institute of Technology, Boston, Mass., on Wednesday, June 5, 1901, and continued the 6th and 7th. The President, Dr. John F. Dowsley, occupied the chair and called the meeting to order. Following is a part of the proceedings of the sessions.

*Dr. Andrew J. Flanagan.*—The committee extended to me the invitation to read a paper before this meeting, perhaps a ten-minute paper, and that the one thought predominant in it might be of some theoretical interest but also of practical interest. With that thought in view, I have prepared a paper, and shall ask for about ten minutes of your time this afternoon.

### TRUE PROFESSIONAL LIFE A FINE ART.

BY DR. ANDREW J. FLANAGAN, SPRINGFIELD, MASS.

This may be said to be the epoch in dentistry of what might be termed "higher education;" accepting this as a fact, we are simply in a race which seems to pervade all life and thought, therefore apace with the general world's activity and advancement. The tireless activity on the part of man may truly be said to be the source of all good. The pessimistic have said it was the source of all evil. Be that as it may, the moral philosopher may well ask, Has the higher life kept apace with the higher education, or is the higher life a thing of the past in this the strenuous age? Let me state in the beginning that the word professional is used in its broadest sense, and that art to me signifies the application or use of knowledge and skill to that which we will term the immaterial and beautiful, a something beyond the worldly.

The essayist has been for seven years an observer and participant in many dental meetings, and naturally has had an insight into dentistry through the individual membership; it is perhaps best to say at once that the ideas gained were of a varied and interesting nature—from my point of view. If I have heard and understood aright, our calling could be said to be divided into two great camps, the practical and the ideal, or, as the other fellow says, the impractical; searching for a true understanding of their meaning has brought forth this: the practical means it is the easiest way and has the best financial returns to himself; the ideal is the sum total of all that is opposed to this. The very practical man is likely to be the fellow that says, "I am not in dentistry for my health." You have all seen him and, above all, heard him. We have men who believe in the usefulness and mission of the various schools, colleges, dental laws, boards of examiners, and societies; and arrayed against them, the one who believes and even knows he can teach our calling better than any college; the dental law is a farce, and to prove it without a doubt he tells you about the number of charlatans "practising right in his own town, and if it allows them to live, what good is it?" boards of examiners are without use, "because how else could the fakir practise?" the societies are simply "mutual admiration associations," and give little practical help. We must not forget to mention the carping critic and the pessimists,—"everything is going to the dogs kind;" and last, but by no means least, the member who takes everything in hints and knowledge from his fellow, and never gives anything in return; in other words, the first-class robber. Many, many more could be mentioned, but the foregoing symptoms are so marked and pronounced that you have long before this arrived at the diagnosis, and it would be a waste of valuable time to continue down the long and varied list.

The human mind's capacity for self-deceivement is without limit. We are prone to think our troubles worse than the other fellow's; arrayed against this last is the saying, "The easiest person to deceive is one's own self." Are we not deceiving ourselves when we claim that the colleges, State boards, State laws, and societies are in any way related to certain dishonest and unethical conditions of the present? The good book says, "The poor you have always with you," and without sacrilegious intent, we could truthfully state, the fakir you have always with you. He is a product of no special clime or time, and until the millennium arrives you may expect to find him. At times pessimism is to be expected, and the strongest—in severe moments—are temporarily cast down. When evil seems to prosper best, remember that "truth alone is strong," and if you have any faith whatever in a divine order you must eventually come back to rest on the verities, and to lose hope or courage because certain conditions are against us for the time being is childish. Have faith, and take consolation in the disposition of the public to do justly where they understand the facts.

In discussing the virtues and evils of our surroundings in general, and our calling in particular, there is a tendency to forget fundamentals, the lack of which means retrogression in any walk of life, but the possession of which permeates and refines all life. These fundamentals have been from the beginning, and woe be to dentistry when they are forgotten. They are the beginning or the kindergarten of all true professional life and the want of which no institution or society can make good. To many eyes these wear a homely garb, are commonplace and unromantic. But they are the very stuff of true professional life, the heart of real power and action, the basis of enduring happiness and peace, of sincerity in speech, of honesty of deeds, of purity of purpose, and of kindness from principle and not from mere transient impulse. They alone ennoble life in every sphere. By accident of birth and circumstances one may arise to high positions, but only by the having of these fundamentals is one worthy of honor. Riches, rank, fame, and learning are mere incidents; it matters little whether we win them or not; these fundamentals are a prize open to all and are that end which fulfils and dignifies life. These fundamentals of every-day and professional life are those qualities which are so beautifully summed up in the homely phrase, true manhood.

After all, what a man is in dentistry resolves itself back into what he is as a man. Before any one is a practitioner of our beloved calling he is a man, and what he is as a man will determine his character in all the departments and relationships of dentistry. Education is but a training of the mind; manhood is but a training of the soul. What a noble combination! a combination which would have made our late good Dr. Garretson to exclaim, "the God-man." The true professional life of the future must be developed along these lines, and then truly can we call it a fine art. We shall then have a refined taste, ear open to appeals, a heart respondent to all necessity, gentleness yet firmness of manners, knightly courtesies, candidness of expression, transparency of motive, nobility of ideals, optimistic by nature—our presence bringing sunshine, greeting, and good cheer; last, but by no means least, "charity towards all and malice towards none." What our French brethren call the "esprit de corps," and we the "code of ethics," will need never to be printed, for each will then be a veritable personification of that true and only code of ethics, manhood. In closing, let me quote to you a few lines of an unknown author, an author which to me has the choicest fragrance of Faith, Hope, and Charity in professional life.

"'Tis weary watching wave by wave,
And yet the tide heaves onward;
We climb, like corals, grave by grave,
That pave a pathway sunward;
We are driven back for our next fray
A newer strength to borrow,
And where the vanguard camps to-day
The rear shall rest to-morrow.

"Though hearts brood o'er the past, our eyes
With smiling features glisten;
For lo! our day bursts up the skies,
Lean out your souls and listen:
The world is rolling freedom's way,
And ripening with her sorrow;
Take heart! who bear the cross to-day
Shall wear the crown to-morrow."

A banquet was held at the Hotel Brunswick at 6.30, where covers were laid for one hundred and thirty-five. After a very enjoyable time the President arose and in the following manner introduced the speakers of the evening.

*President Dowsley.*—Fellow-members, ladies, and gentlemen, as the presiding officer this evening the very pleasant duty is incumbent upon me to welcome you here on the occasion of the thirty-seventh anniversary of the Massachusetts Dental Society. The theme selected for discussion is a subject in which we are all deeply interested, and we are fortunate, indeed, in having with us as our guests gentlemen whose life work has been in this direction. Notwithstanding the unparalleled progress made in dentistry and dental education within the last half-century, the resources in this direction are far from being exhausted, and at no period has greater progress been made than is being made at the present time. Therefore, any institution of dentistry which would maintain pre-eminence must be alive to the needs of the people, the demands of the profession, and the scientific developments of the day. In other words, it must be progressive in the fullest signification of the term. But I must not tax your patience, for of course I know how anxious you are, as, indeed, I am too, to hear from the very distinguished gentlemen who have honored us with their presence. It therefore gives me great pleasure to present to you, as the first speaker of the evening, President Elmer H. Capen, of Tufts College. (Applause.)

As Dr. Capen rose to respond, the musicians sang the following college song, which belongs to Tufts College:

"While our colors float above, we will cheer the home we love,
Where already we have lingered long;
Let our spirits rise to-night, let our hopes be warm and bright,
We will banish sadness with our song.

"So we hail the brown and blue, with a love forever true,
We will fling our banner to the sky;
But the world of storm we crave, so we skim along the wave,
'Mid the tempest cheerfully we sing, we sing."

*President Capen.*—Mr. President, ladies, and gentlemen, I am glad to be able to sing the "Brown and Blue" with you. I certainly appreciate the compliment of an invitation to this banquet, and have enjoyed myself since I came until this very present moment. I should fail to express my real feelings if I did not say that I am under not a little embarrassment in rising to respond to the call of your president. I used to get on very well with public speaking when I had only ministers or people of general literary culture to address. But since I began to give attention to, and become in some sense responsible for, medical and dental education, I have been brought into strange fellowship,—very delightful fellowship I am bound to confess,—and somehow it almost paralyzes me when I rise to address gentlemen who are trained in mysteries and technicalities that are wholly foreign to my own field of knowledge.

I recall a story of a very good navigator, a mate of a ship, who occasionally imbibed more than was good for him. One day when he was in this condition, the captain wrote in the log: "Mate drunk." On his return to duty, the mate asked the captain why he wrote it. "It is true, is it not?" replied the captain. "Yes," said the mate. "Then let it stand," was the captain's answer. A little while after, the captain found that on a certain day the mate had entered, "Captain sober to-day." Naturally he called the mate to an account. "It is true, is it not?" said the mate. "Of course it is," said the captain. "Then let it stand," was the mate's reply. The bearings of this story, as Jack Bunsby would say, is in its application. So far as this occasion is concerned, if I should succeed in making a good speech, it would not prove that such is my usual habit, while if I should make a poor one, as I fear I shall, you would not, I regret to say, be warranted in the conclusion that I am a successful public speaker.

I might, perhaps, save myself from the possibility of failure by dealing in generalities. I might speak on the importance of the dental profession, the opportunities it presents for service to humanity, and how much it has contributed to health and happiness, and even to the prolongation of human life. There is just one phase of the matter which I am justified in attending to, and that is the immense and increasing interest which has now been awakened in preparation for the dental profession. As your president has just said, there is a demand among dentists for a broader and more scientific education. Dentistry shares in the demand that is now made upon all the professions for a broader as well as a more accurate knowledge and a more extended and thorough preparation.

My relation to dentistry and medicine, and, indeed, all the specialties, is that of an educator. When it was first proposed that Tufts College should assume control of the Boston Dental College, for myself I thought, and in this I am sure that I voice the sentiment of the trustees of Tufts College, that we could render to education in general, and to dental education in particular, a distinct service by such a course. Here was an institution that had an honorable history, that had done good work throughout the years of its existence, that was progressive in its spirit, and striving to meet the exigencies of the times. It could be assisted in its efforts by coming into relation with some institution in which medical education was given. It did not seem to me that we had any option in the matter. That is how Tufts College came to be engaged in the business of dental education. I ought to say, further, that since I began to give attention to these things and broaden my acquaintance with them, I have been impressed with the fact that the members of your profession are alive, that they are looking ahead and reaching out in all directions for whatever may make them stronger and more efficient, realizing that they cannot fulfil the ends of their calling without laying the broadest possible foundations and doing everything in their power to secure a scientific training and attainment.

I have recently read, with much satisfaction and, I trust, edification, the report of the recent meeting of the National Stomatological Society. It is true, I could not understand all that I read, and I do not claim to have any ability to pass judgment upon what I read. I cannot say whether the various papers record substantial contributions to the science of dentistry in its different departments of application and research, nor do I think *this* a matter of the highest importance. The thing of highest significance is that a body of men representing the dentists of the United States should come together and seriously consider, in a spirit of true scholarship, the great questions by which they are daily confronted in their professional experience; that they should not only be pressing forward for the solution of these problems, but that they should be seeking most earnestly for the practical equipment which would make them safe, trustworthy, and successful practitioners. It seems to me this is a very great encouragement, a most hopeful sign.

Moreover, there is abundant opportunity for important achievement in this direction. In our experience, thus far, we have found not only a popular demand for better-educated dentists, but there is a readiness on the part of the young men who are looking towards dentistry as a profession to submit to every demand that may be made upon them by way of preparation for their work. It is just here, also, that you gentlemen, in all the States of the United

States, may exert a tremendous influence upon the young men of your profession by holding up the noblest ideal and by insisting upon it that they shall take all needful time in their preparation. You should see to it that they acquire something more than technical knowledge and manipulative skill. Their work is related to the whole field of human health, and they are to hold their places as educated men among other men of liberal training. There must, therefore, be some space for broad and general training, and it should precede the period of specific preparation. You cannot erect a mammoth structure upon a single pile. You must have a proper foundation for it. You must put that in, moreover, beforehand. It may be conceivable that the Cologne cathedral could be built from the top downward. But just think of the enormous increase in the expense of time and money. Yet, alas! this is the thing that we are often doing in education. If you were to make a candid confession, many of you, no doubt, would say, "I began my education at the wrong end. I got into the profession by a short cut, and ever since I have been building from the top down." You should insist, therefore, for those who are to stand in your places by and by, that they should take ample time for preliminary education, and that they should also make their specific training full and thorough.

Speaking for Tufts College, I may say that we have definitely in mind, at least, a high-grade high school education as a requisite for admission both to our medical and dental schools. We would not let these schools drop below the level, in this respect, of our State normal school. Harvard University, to be sure, has gone much farther than that in its medical school by requiring a college education, or its equivalent, as the condition of admission. I do not know whether it is their intention to apply the same rule in their dental school. But I think we must all admit that this is the ideal thing, and we can, at least, work towards it, in the hope that the time may come when nearly every dentist will have had a college education before seeking his preparation in a dental school.

I may be permitted to say, before I take my seat, that we shall open the next school year in our new building on Huntington Avenue. We have not had much money to expend in fine architecture, but we have sought to secure all the requisite facilities for good working laboratories and lecture-rooms. I think you will say, when you inspect the building, that we have been successful in this. We shall hope to see you all at our opening exercises on the first day of October next.

*President Dowsley.*—I would like to call the attention of the ladies and gentlemen present to the fact that you will find the words of the songs for this evening on the menu, and I know that we have a great many singers present among the ladies and gentlemen, and I would like to hear from them all in this part of the exercises.

The next name on the programme is that of a most distinguished gentleman from Philadelphia. I say distinguished, because wherever dentistry is taught, wherever it is read, his name as a writer and an educator has become a household word. I know that every one of you will regret very much that he is not present, and I had hoped up to the last moment that he would be here, but I received a letter from him this morning telling me of the impossibility of his being here.

I regret this very deeply, first, because I did not want to disappoint you, and secondly, because I am very desirous of hearing him discuss the subject of dental education, but unfortunately he cannot come because it is impossible for him to be in two places at once. In the letter I received this morning he goes on to say, "I want to compliment you on the beautiful programme which your committee has gotten up. Much as I dislike to say it, I think that Boston is on top this time. Although I cannot be with you in person, I am with you in spirit." Of course, you all know I refer to Professor Kirk, Dean of the Dental Department, University of Pennsylvania, and editor of the *Dental Cosmos.*

The University Hymn was then sung as follows:

"Our fair Alma Mater, oh, strengthen her days,
To send forth forever true sons to her praise;
Oh, widen her borders, extend her fair fame,
And let all the glory redound to her name."

*President Dowsley.*—The next speaker comes to us from the South, from the State which was the home of dentistry. The college he represents is where dentistry had its birth. The position he occupies, as professor in this particular school, is an important one, since upon him has fallen the mantle of the pioneers of dental education. I take exceeding pleasure in presenting to you Professor B. Holly Smith, of the Baltimore College of Dental Surgery.

Upon Dr. Smith's introduction the following song was rendered:

> "Thou wilt not cower in the dust, Maryland, my Maryland.
> Thy beaming sword shall never rust, Maryland, my Maryland.
> Remember Carroll's sacred trust; remember Howard's warlike thrust;
> And all thy slumbers in the dust, Maryland, my Maryland."

Response to toast by Dr. B. Holly Smith, of Baltimore, Md.:

Mr. President, Ladies, and Gentlemen,—I assure you I feel grateful to the one who arranged this programme for tincturing my introduction with the patriotic sentiments that swell in the hearts of all loyal Marylanders when this song is sung. ("Maryland, my Maryland.") This State love and pride is a fit parent to college love and pride, and college work is our subject, I believe.

Judging from the papers and reports of discussions to be seen in the journals, every one talks and writes about education. Such varying and widely different opinions prevail as to force one to accept them as individual and not class opinions.

Upon three points perhaps, a large majority agree: first, that preliminary requirements should be increased; secondly, course extended; and thirdly, methods of instruction improved. Many contend for a preliminary qualification equivalent to a college or university degree. I insist that the course of instruction in the average college is not the one best fitted to prepare a student to make the most of his opportunities in a dental course. Too often he is warped and dwarfed by the methods used, and fails of a symmetrical development. What is gained is gained at too great a sacrifice of time, the student having the edge taken off his ambition, and approaching the special preparation for his life work with an absence of initiative and a *blasé* temper. But beyond all he has to begin his mechanics with absolutely untrained hands, at a time of life when motor impulses are generated and execution accomplished with greater difficulty than at an earlier period.

Professor C. F. Warner, before the Valley District Dental Society of Massachusetts, says, as to preparation for the study of dentistry, "Certainly the student ought to have cultivated a certain general command of himself, both of his intellectual and manual powers. He should have a knowledge of the fundamental principles of physics and chemistry, physiology and hygiene, a good working knowledge of English, a working knowledge of Latin, but in addition the ability to observe, discriminate, reason, and co-ordinate muscular movement under the direction of the will. An educated man is one who has cultivated all his powers, and can direct them to the highest in whatever field his work may be."

You in Boston who are interested in preliminary training do not realize your opportunity. Why not secure the arrangement of a course of instruction in the Institute of Technology, which might be recommended as the ideal preparatory course for the dental student?

As to increasing the number of years in the dental course, I am not disposed to favor that, because I think it an unnecessary imposition upon the student. The student who cannot be prepared to begin the practice of dentistry in three years ought to select something else for his work. Of course, it is not contended that all who enter the race will make men qualified for our profession; a small per cent. of men who attempt anything fail; we see college graduates filling positions out of all keeping of their supposed preparation, such as conductors on our street-cars; but after a proper preliminary course and faithful performance of his duties in a good dental college for three years, the average student is in a position to become at no distant day a professional man of liberal education; all the more useful as a member of the community because he is a man of action and not a dreamer.

In the matter of improvement in the methods of instruction, too much cannot be done. My opinion is that the dental course should be entirely separate and distinct from the medical one. Where students of medicine and dentistry attend the same lectures, the teaching is for the medical and not for the dental student. The latter is forced to make his own selection out of the material offered him, of that which is vital to his work. Not only this, he is invariably discredited when examination day comes, and the thought is unavoidably present in the teacher's mind that he is *only* a dental student. Let the teacher teach general materia medica, physiology, chemistry, therapeutics, etc.; but what lecturer at a medical school stops to emphasize the importance of the dental application in his teaching? When this plan is pursued, four or even six years would be necessary perhaps. It was not all a joke when the president of a university said, not long since, that to properly educate one man in every specialty in medicine would take twenty-eight years.

Let the dental student have his instruction in a class to himself, by a teacher who studies his subject from a dental stand-point. Above all, Mr. Chairman, let the teacher himself be well qualified. We should select our teachers because of their special fitness rather than because of their accessibility.

" Give us men!
Strong and stalwart ones!
Men whom highest hope inspires,
Men whom purest honor fires,
Men who trample self beneath them,
Men who make their country wreathe them,
As her noble sons,
Worthy of their sires!
Men who never shame their mothers,
Men who never fail their brothers,
True, however false are others.
Give us men, I say again,
Give us men!"

*President Dowsley.*—If this meeting was held at the North Pole or in Central Africa, perhaps I would feel obliged to say a word by way of introduction in presenting the next speaker, but all that I feel it is necessary to do here in Boston is simply to mention the name of Professor Eugene H. Smith.

As Professor Smith rose to respond, the musicians sang " Fair Harvard."

" Fair Harvard, thy sons to thy jubilee throng,
And with blessings surrender thee o'er,
By these festival rites, from the age that is past,
To the age that is waiting before.
Oh, relic and type of our ancestors' worth,
That has long kept their memory warm!
First flow'r of their wilderness, star of their night,
Calm rising thro' change and thro' storm."

*Dr. Eugene H. Smith.*—Mr. President, ladies, and gentlemen, I think it was about a year ago that I met our worthy president on the street corner, and there we discussed in an informal manner, for about a half-hour, matters pertaining to education, dental education in particular. Our worthy president claims that during that conversation I made a promise to be present at this meeting and address you. I do not remember it, but I am willing to assume that he was right. It seems hardly necessary that another Smith should speak after such brilliant entertainment as we have had from Baltimore. He has shown the fine humor of the family, and I will not endeavor to go into that part of it. But to be more serious, Mr. President, last month, under the auspices of the Dental Department of the University of Maryland, there was unveiled a tablet, embracing busts in high relief of two pioneers in the field of dentistry,—namely, Dr. Horace B. Hayden and Dr. Chapin H. Harris.

It was in 1837 that Dr. Hayden gave to the world the first course of Scientific Dental Lectures, and three years later, in 1840, Dr. Chapin Harris established the Baltimore College of Dental Surgery.

Since that day to the present time there has been carried on, through the medium of societies and through dental journals, a discussion, bitter at times, on the subject which is before us this evening,—namely, dental education.

It has been discussed under various headings, such as "Is Dentistry a Specialty of Medicine?" "The Status of the Dental Profession," "Should a Dentist be first graduated in Medicine and afterwards obtain the Dental Degree?" and "Should the Dental Degree be abolished?"

It is a matter of dental history that Dr. Harris sought first to establish a Chair in Dentistry in the Medical Department of the University of Maryland. Failing in that the special school in dentistry had its beginning.

To many minds that step was a grave error, and since that time the multiplication of dental schools and the rapid and unprecedented advancement of our profession along the lines then laid down has seemed to these same good people an educational blunder of stupendous magnitude; while others feel, and it seems to me with good ground for such feeling, that that step of Dr. Harris was the wise beginning of a system of dental education, which has given to the world the skilful American dentist, a benefactor of his race.

The dentist of to-day is more the product of the dental school than he is the product of the medical school. It is true that we are not satisfied with that product, neither were we any better satisfied with the product of the medical school during the time of a similar cultivation.

I sometimes think when I listen to or read the statements of men in our profession regarding a thorough medical education for dentists, and the doing away of the special dental degree, that they cannot be in touch with the present method of medical education, or with the present method of dental education as exemplified in our best schools.

So far as I am able to judge, these statements come largely from men who obtained their degrees, be they in medicine or dentistry, in what might be termed the olden time, and have little or no conception of the present status of the medical or dental school.

The cry, namely, that the wayward child, dentistry, should at once return to its parent, medicine, comes largely from men who obtained their degrees in medicine when the training meant but little.

This statement of fact does not in any way reflect to their discredit. They live up to the best of their time. It only reflects discredit when they try to make more of it than the facts warrant.

To illustrate, a doctor of medicine practising dentistry made recently this statement: "The papers which we have heard all tend to prove the need of a sound medical education as the foundation of a dental practice. This is not new, but rather a reversion to original conditions. In 1844, when I wanted to study dentistry, I found no reputable dentist who would accept me as a student unless I would first study medicine, and I, therefore, spent three years getting a medical degree before entering upon the study of dentistry."

Now, this statement of the good doctor is misleading. In 1844 a student on his way to his medical degree was not obliged to spend three years in medical study. The conditions required at that time were these:

No entrance examinations. Attendance on medical lectures two winter terms of five months each; and the second term's work was largely a repetition of the work of the first term. The final examinations were oral, the results decided by votes, a majority of which passed the applicant for the degree.

Now, I do not say but what he spent three years in getting his medical degree, but if he did, he was either conditioned or else he spent a longer time than was absolutely necessary.

The question that echoed through the ages and satisfied the past—namely, "How much do you know?"—does not meet the requirement of to-day. There must be coupled with it this question, so pregnant to our profession,—namely, "What can you do?"

I have been unable to find in the writings of those who would abolish the dental school and its consequent degree any feasible plan for the absorption of the dental student into the medical scheme that does not provide for less technical dental training than the dental student now receives. To this I am strongly opposed. My observation during the past few years leads me to think that the M.D. degree already covers too many specialties in medicine, and that it would be most unwise for our profession to nestle under its wings.

Those who would like to see the degree in medicine for dentists, and at the same time not sacrifice the necessary technical training, think they see in the elective system a means to this end, and cite this system as applied to the collegiate course in support of their views.

It seems to me that the comparison is erroneous, and that the elective system in medicine can have but a very limited application, and, therefore, would afford but little time-saving from the present requirements for the medical degree.

If, however, an elective scheme of medical and dental training can be so arranged that the dental specialist may be a graduate in medicine, I would still hold to the dental degree, for I feel that if the evidence of special training were thrown aside, we would suffer in our ranks men who, while studying for the medical degree, had elected but few of the special dental subjects, and had also neglected the training in dental technic.

We hear much regarding the status of our profession as compared with other professions, and the opinion is advanced that we should save time on the dental side of an elective system by eliminating the mechanical laboratory element which now enters into dental training, and at the same time advance our professional standing, inasmuch as the mechanical side detracts from professional worth.

To my mind this is a foolish and weak argument. The status of a profession or calling is determined, I think, by the quality of the men who enter it, and that quality must, in our profession, be determined by education and skill.

I believe that a mechanical education is as much a part of the higher education as are many of the studies termed culture studies.

I believe with Sluy, the Swedish writer, who affirms that "Mechanical education insures the integral cultivation of all the faculties and all the aptitudes which make up the complete man."

I am a believer in the dental schools. I believe that the standard of requirements for entrance to the dental schools should be steadily and rapidly increased until, at last, every dental department of a university becomes a graduate school.

I believe that the course of study should cover four years of nine months each.

I believe that a part, but not all, of a general medical education should be required of the dental student.

I believe that the technical training should be more than he now gets in the best dental schools, and, believing this, I shall labor to that end, for I fear that if we sacrifice the dental training, as it is best understood, for many of the medical branches that we do not need, we shall be left weak and sad, and say with Faust,—

> "I've now, alas! Philosophy, Medicine, and
> Jurisprudence too, and to my cost
> Theology, with ardent labor studied through;
> And here I stand, with all my lore,
> Poor fool, no wiser than before."

*President Dowsley.*—I have had the pleasure on previous occasions of listening to the gentleman whose name is next on the programme, and all that I need say is that I can vouch for him. He comes from a progressive college, being the second in the country that is about to adopt the four years' course. I have great pleasure in presenting to you as our next speaker Professor Harold Williams, of Tufts College.

The song entitled "Charlie Tufts" was then sung as the Professor rose to respond.

*Dr. Harold Williams.*—Mr. President, ladies, and gentlemen, I am very much disturbed in my mind by something Dr. Holly Smith has said. I allude to his fear of foreign importations in dental education, which he likens to the importation of the gypsy moth and the mongoose. It reminds me of a story. A Londoner of inquisitive disposition was once travelling in a railway car. Opposite him sat a man who had a very curiously shaped bundle. After a good deal of uneasiness, the Londoner finally turned to the man and said, "Would you excuse me, sir, if I asked you what you had in that bundle?" The man looked at him a moment, and then answered that he had a mongoose. After another period of uneasiness, the Englishman once more said, "Would you excuse me if I

asked you what a mongoose was?" "It is an animal which is used in the Western islands for killing snakes." "Excuse me, sir," said the inquisitive gentleman again, "but would you mind telling me what you are doing in London with an animal for killing snakes?" The man replied, "I am taking it to a friend of mine who has delirium tremens." "Oh, sir," said the Londoner, "those are not real snakes." "No," replied the man with the bundle, "but this is not a real mongoose." It seems to me that these fears of Dr. Smith's are not real enough to be alarmed about.

But now, getting down to the business of the evening, there is not very much left for me to say on the subject. In regard to the four years' course, many of you will remember that my connection with dental educational affairs is a recent one. But in spite of the shortness of the time, I have studied and looked at this matter from every point of view, and it seems to me the only thing for us to do is to lengthen the dental course. Under the present system the best schools may be divided into two classes. One class of these schools is where a knowledge of science is taught at the expense of dentistry, and the other is where dentistry is taught at the expense of science. Neither of these systems has been satisfactory up to the present time. We have good mechanical dentists who lack in scientific knowledge, and who consequently cannot give to their patients the modern applications of science which they have a right to demand. On the other hand, I find a number of dentists who learn the major part of their mechanical and operative work in the early years of their practice. Both classes are scantily equipped, and it seems to me that the only way that we can get around this question is to increase the course and make it a full four years, as in medicine, which will give the dentist a little more of the scientific part of dentistry than he gets in the present school without curtailing his purely dental training. These are briefly my views on dental education, and I am pleased to say that the Tufts College Dental School is going to adopt the four years' course. This change will go into effect in 1902.

Now, there are one or two subjects which have interested me very much, indeed, since I have been connected with the dental school. In regard to these subjects, let me say that it is not the education of the dental student, but the education of the public as to the necessity of dentistry. I wish I could have heard Dr. Hopkins's paper this morning. In modern times preventive medicine, the prevention of disease, is regarded as one of the most important branches of medicine. Dentistry is a branch of preventive medicine, and it is of great importance to educate the public as to its vital necessity. One of the subjects to which I allude is the question of dental infirmaries which supply free treatment for the poor. There are in Boston two great institutions of this nature,—our school and the Harvard school. They receive annually less than fifty thousand patients,—in our school thirty thousand and in the Harvard school twenty thousand. Take Boston as a centre of population, with a radius of fifty miles, and it is the second largest centre of population in the United States. We have in Boston an immense number of medical charities, and a vast number of people are charitably treated in these medical institutions. One hundred and fifty thousand people who annually receive free treatment from the medical institutions of Boston will be a conservative estimate. When you compare that one hundred and fifty thousand sick people who receive free medical treatment with the fifty thousand well people who receive dental treatment, it gives you an idea of how slightly the importance of dentistry is understood and what an enormous field is open to the dental profession. It seems to me that this is a subject that should appeal most strongly to the members of the dental profession. I am afraid that dentists are failing in that paramount duty of pointing out to their patients and to the laity the importance of dentistry in the way of preventive medicine. It seems to me almost incomprehensible that in this great city no public provision has been made for these two great institutions, and that up to the present time no benevolent support or endowment has been made for these two great charities which are fulfilling one of the most important functions in our advanced civilization.

The other subject to which I allude is the fact that this Dental Society is not leading the way in regard to the dental examination of the public school children. The Massachusetts Medical Society and the members thereof, with the Boston Board of Health, first instituted the medical examination of the public school children, and it seems to me very remarkable that members of the Dental Society have not inaugurated a similar crusade for the examination of the children in our public schools. Such an examination would be the means of preventing an enormous amount of disease, and would be an inestimable boon to the community. This has been recommended by Dr. Grady, of Baltimore, and I hope that we shall soon see the dental profession leading the way along these lines in Boston and its vicinity.

*President Dowsley.*—It is something over thirty years since the Dental Department of Harvard University was established. In this connection I would say it has been said that there is always something rather pathetic about the infancy of an institution. One of the pathetic things is concerning the men who have put their lives into the infancy of these institutions. I know several men eminent in their callings who have put their lives into the infancy of this school. We have with us to-night the only surviving member of that corps of professors, and it gives me great pleasure to present to you Dr. L. D. Shepard, of Boston.

Upon Dr. Shepard's rising to respond, the musicians sang the words of "Auld Lang Syne."

*Dr. L. D. Shepard,* Boston, Mass.—Mr. President, ladies, and gentlemen, the introduction of your president has touched a chord in memory, to which perhaps I may be allowed to refer for a moment. The first president of this Society, the late Dr. N. C. Keep, in his annual address suggested the advantage of instruction in dentistry in connection with the Harvard Medical School. There was then no dental school in New England. In compliance with the suggestion, a committee, consisting of Drs. Keep, Rolfe, and myself, was appointed to confer with a similar committee from the Medical School. The two committees, after many conferences extending over several months, elaborated a scheme for a dental school, substantially as it has existed ever since, and which was the first in the world in which dental instruction was carried on in connection with a university. When the Dental Faculty was appointed it consisted of Drs. Bigelow, Bacon, and Holmes on the medical side, and of Drs. Keep, Hitchcock, Moffatt, and myself. Of these two committees of conference, and of the first dental faculty, I stand here, as your president has told you, the only living representative. My many years of connection with the school and my association with these devoted men who have gone before has made the memory of the past very dear to me. Graduating also, as I did, from the Baltimore College of Dental Surgery before the great war of the rebellion was started, I may in a certain sense be said to have come down to you from a former generation.

Under these circumstances, what place should a retired veteran have in the councils of the active generals of education? You, gentlemen, deans and professors, who have preceded me, have resting upon you the responsibility of deciding all questions. You feel the weight of that obligation. One in my position possesses the advantage of looking over the ground, unhampered by a vital interest in present problems, and with a greater knowledge of the history of the evolution of dental education than any young man can possess.

In early days, nineteen out of twenty entered upon practice with only an office training, frequently of a few weeks only, the college education being the voluntary choice of the few. To-day, thanks principally to dental laws, and happily for the college, the profession, and the public, the college education is a necessity for all.

I do not agree with the statement that the profession is crowded, or that it is likely to be for many years. While the rich may be growing richer, there is a constant increase among the masses of the ability to pay for greater comforts and luxuries, and so a constant broadening of the field for dental services. This is aided also by the more general appreciation of its needs and benefits.

In view of the wonderful discoveries and inventions of recent years, such as the telephone, the phonograph, the X-ray, and now wireless telegraphy, it would be hazardous for any one to deny that any invention is possible. The practice of our art may become unnecessary, but at present I see no prospect of such an improvement in nutrition as to insure tissues of so much greater resistance to attack, or so much simpler preventive or restorative measures as to make the operative dentist less a necessity among the people for their relief and succor. So the needs upon which our work is founded is increased year by year. Although hundreds may be graduated each year, the material upon which the dentist shall work will increase faster than the supply of workers.

I sympathize with all the progressive movements tending to the elevation of the profession, the broadening of the field of instruction, and the more thorough placing of our calling among the learned professions. I agree that the more general and thorough an education a dentist may have the better dentist he is, provided always that he acquire this at no lessening of positive manipulative skill. It has been a great satisfaction to me that my son has been willing to patiently study four years in college, four in the medical school, and two in the dental school, when, instead of these ten long years, he could have graduated from the dental school in three years, and I am happy that I was able to give him the advantages he was willing to take. I believe such a course is desirable when the conditions are favorable.

But remember we live in a strenuous age, never more so. Competition was never so keen, and the struggle for the survival of the fittest never so earnest. So such a course of instruction, however desirable, is a luxury for the few, and it is not within the limits of possibility for forty-nine out of fifty of the ambitious and promising young men who are to take our places and be useful to suffering humanity.

How long or how short a course must we insist upon for these forty-nine students? From my experience as a student and teacher, and my observation for forty-odd years of schools and practitioners, I would insist upon certain attainments as imperative, and upon others as desirable in addition to the imperative.

The fundamental imperative attainment is manipulative skill. In the olden times a large part of this training was received by the student in a dental office, remaining perhaps for years, where he acquired a knowledge of materials and their uses and skill in performing various operations and in the control of the hands. He then entered the college for theoretical training and to acquire a broader knowledge of technics than the private office could give him. In modern times the student comes generally to the college from the high school, and entirely untrained in either theory or practice. Under the head of manipulative skill I include all that is generally understood to be the office work done on or for a patient by a dental mechanic, in distinction from that part which is founded upon science or medicine. Under this head comes a knowledge of physics; the physical properties of materials, and how to best manipulate them; the law of forces and their application; the trained eye which can accurately estimate distance, direction, form, etc.; the reasoning mind, which, informed by the trained eye, can determine what is needed, and direct the hand in the doing; and the skilful fingers, which under such intelligent direction can, with accuracy and with the least waste of effort, perform all the work required to produce the finished and perfect result.

All surgery in its etymology shows its handwork. The practical skill of no specialty of surgery requires as much time or as many repetitions of the doing of each act as dental surgery. I cannot take time to elaborate this point, but I think it is true. There are a few individuals in every one hundred who are so gifted that they can acquire this knowledge and skill in a short time, but I am convinced that the great majority of those seeking to be dentists will require at least two years of nine months each to gain sufficient skill to be entitled to graduation.

May I here interpolate the remark in connection with the project to lengthen the course of study to four years, that it is my opinion that under present conditions, when the student cannot as in former times earn anything in vacation, any school whose year of study is less than that usual in technical schools, like the Institute of Technology in Boston, is defrauding the student. Vacations are for rest, and should be, in justice to the student, of such length only as will serve that end.

So much time being required for preparation for work, how much shall be given to theory and science? Can we demand in this practical age as a preliminary to fit a man honestly and honorably to earn his living more than he may need to thoroughly equip him? He cannot have too thorough a knowledge of the anatomy of the head, the ramifications of the fifth nerve, of the eighth, also of the fourth and sixth, their mutual dependences and reflexes. He should know the thorax, respiration and blood circulation, and the digestive organs. But why should he waste time on the names and articulation of the bones of the foot, or the origin and insertion of the muscles of the legs? In a word, the dental student should have a special course in anatomy, more thorough in some respects than that now given in any school, and carefully eliminating the superfluous. Less criticism on other subjects applies to the schools as at present conducted. I would include in the course general and special physiology, pathology, therapeutics, chemistry, microscopy, bacteriology, and other special subjects of use in dental medicine and surgery. I do not think these subjects can be taught in less than two years. So we come to the conclusion that the college of the future must occupy all the time of the student for four years. I need hardly add that I do not consider in this scheme the order of the various studies and work, except that I do believe that some test of the mechanical talent of mind and hand should enter into the curriculum of the first year.

I have had a large acquaintance with dentists in this country and in Europe. I regret to have to admit that, as a rule, those of great learning have been poor operators, and that many with little learning have been a blessing to the community in which they labored. We all mourn the friend who has just died in St. Louis, full of years, of dental honors, and of grateful remembrances. He was an ideal dentist, the noblest Roman of them all. He did not know much of medical science, and could not be called a learned man, but during his long life he kept in view the one object of the perfection of his operations, and how to do the greatest good to his patients. His hand was open to every brother dentist. In the societies and in the clinics he was a teacher to thousands, stimulating all who came within his magnetic influence to higher ideals and more finished execution.

I have a confidence that in the future learning and skill will go hand in hand. In closing, I will reiterate my conviction that, however much a man may know, whether he may be able to talk science with Huxley, or sociology with Spencer, he must have the reasoning brain which can investigate, decide, and direct, must have judgment of distance, size, and form, and a thorough training of those wonderful instruments the fingers, before he can perform the beautiful and finished works which will make him a blessing to those who put their trust in him.

(To be continued.)

